# Identification of Altered Metabolomic Profiles Following a *Panchakarma*-based Ayurvedic Intervention in Healthy Subjects: The Self-Directed Biological Transformation Initiative (SBTI)

**DOI:** 10.1038/srep32609

**Published:** 2016-09-09

**Authors:** Christine Tara Peterson, Joseph Lucas, Lisa St. John-Williams, J. Will Thompson, M. Arthur Moseley, Sheila Patel, Scott N. Peterson, Valencia Porter, Eric E. Schadt, Paul J. Mills, Rudolph E. Tanzi, P. Murali Doraiswamy, Deepak Chopra

**Affiliations:** 1 Center of Excellence for Research and Training in Integrative Health, Department of Family Medicine and Public Health, University of California San Diego, La Jolla, California, USA; 2 Department of Ayurveda and Yoga Research, Chopra Foundation, Carlsbad, California, USA; 3 Proteomics Core Facility, Duke University, Durham, North Carolina, USA; 4 Proteomics and Metabolomics Shared Resource Center for Genomic and Computational Biology, Duke University Medical Center, Durham, North Carolina, USA; 5 Chopra Center for Wellbeing, Carlsbad, California, USA; 6 Department of Family and Preventive Medicine, University of California San Diego, La Jolla, California, USA; 7 Infectious & Inflammatory Disease Center, Sanford Burnham Prebys Medical Discovery Institute, La Jolla, California, USA; 8 Icahn School of Medicine at Mount Sinai, New York, New York, USA; 9 Genetics and Aging Research Unit, Department of Neurology, Massachusetts General Hospital and Harvard Medical School, Boston, Massachusetts, USA; 10 Departments of Psychiatry and Medicine and the Duke Institute for Brain Sciences, Duke University Health System, Durham, North Carolina, USA

## Abstract

The effects of integrative medicine practices such as meditation and Ayurveda on human physiology are not fully understood. The aim of this study was to identify altered metabolomic profiles following an Ayurveda-based intervention. In the experimental group, 65 healthy male and female subjects participated in a 6-day *Panchakarma*-based Ayurvedic intervention which included herbs, vegetarian diet, meditation, yoga, and massage. A set of 12 plasma phosphatidylcholines decreased (adjusted *p* < 0.01) post-intervention in the experimental (n = 65) compared to control group (n = 54) after Bonferroni correction for multiple testing; within these compounds, the phosphatidylcholine with the greatest decrease in abundance was PC ae C36:4 (delta = −0.34). Application of a 10% FDR revealed an additional 57 metabolites that were differentially abundant between groups. Pathway analysis suggests that the intervention results in changes in metabolites across many pathways such as phospholipid biosynthesis, choline metabolism, and lipoprotein metabolism. The observed plasma metabolomic alterations may reflect a *Panchakarma*-induced modulation of metabotypes. *Panchakarma* promoted statistically significant changes in plasma levels of phosphatidylcholines, sphingomyelins and others in just 6 days. Forthcoming studies that integrate metabolomics with genomic, microbiome and physiological parameters may facilitate a broader systems-level understanding and mechanistic insights into these integrative practices that are employed to promote health and well-being.

Integrative medicine practices, such as meditation and Ayurveda, are popular but their effects on human physiology are not yet fully understood. Ayurveda, a Sanskrit word that means the ‘Science of Life’ or the ‘Science of Perfect Health’, is the traditional system of personalized medicine from India that emphasizes disease prevention and health promotion. *Panchakarma*, Ayurvedic medicine’s principle cleansing and rejuvenation protocols, incorporates various treatment modalities such as a vegetarian diet, Ayurvedic herbs, meditation, yoga, oil massage, heat therapies, and other specialized treatments that are thought to promote general health and well-being^1^. While numerous studies have examined the health and well-being benefits of practices such as meditation, yoga, Ayurvedic herbs and diet, few studies have examined the effects of traditional medical protocols that employ several of these modalities concurrently in one program. To date, very few controlled studies on *Panchakarma* have been undertaken. Previous studies on *Panchakarma* have reported improved psychosocial outcomes in healthy subjects as well as improved psychological parameters in depressed patients^2^,^3^. However, little evidence has been reported regarding the physiological and metabolic effects of such treatments. Thus, given interest in integrative therapies with the general public and medical community is steadily increasing, there is a burgeoning need for additional studies that investigate Ayurvedic medicine with modern scientific techniques to further elucidate the relevant biological effects and mechanisms related to these practices.

Previous studies on the potential health benefits of meditation, yoga, Ayurvedic herbs and diet have examined a wide variety of impacts; however, the majority of these studies have suffered from underpowered cohorts as well as a small number of measured outcomes and analytes thus limiting the generalizability of findings. For example, a number of studies examining the impact of meditation have indicated clear benefits with respect to blood pressure and heart rate variability but not yet demonstrated a broader systems-based understanding of its impact on human physiology and disease prevention^4^,^5^,^6^. Many studies have focused on the effect of Ayurvedic herbs on human health thus supporting the notion that these herbs may have specific impacts on host pathways implicated in disease initiation and progression^7^,^8^,^9^,^10^. Therefore, there is a need for additional investigations that apply modern scientific methodologies to establish a more systems-level understanding of the underlying mechanisms of Ayurvedic medicine.

The human metabolome represents a dynamic interplay among gene expression, protein expression, environmental factors and well-being. Targeted metabolomics is a powerful approach that combines liquid chromatography and mass spectrometry to simultaneously measure low-molecular weight compounds that are intermediates or endpoints of metabolism. The fields of research employing global studies such as genomics, proteomics and metabolomics offer promising approaches that may assist epidemiologic studies to identify candidate biomarkers for risk assessment of chronic diseases, such as cardiovascular disease and diabetes. Since metabolites represent a rapid response to physiologic perturbations, they offer insights into small molecule signaling and may represent appropriate reporters of intermediary, health, and disease phenotypes^11^. Metabotypes, or metabolic phenotypes, capture net interactions between gene, environment and gut microbiota interactions providing information that can potentially link diverse genotypes with phenotypes^12^.

While much focus has been applied to identifying biomarkers of disease, few studies have focused on defining the molecular signatures of health resulting from integrative medicine practices such as *Panchakarma*. Here we report observed alterations within a biomarker panel targeting 186 plasma metabolites from 5 distinct metabolite classes (i.e. amino acids, biogenic amines, acylcarnitines, glycerophospholipids and sphingolipids) in heathy human subjects following a 6-day *Panchakarma*-based Ayurvedic intervention. *Panchakarma*, which included vegetarian diet and Ayurvedic herbs with potential hypolipidemic action, promoted statistically significant changes in observed plasma levels of phosphatidylcholines, sphingomyelins and others in only 6 days.

## Results

A total of 261 subjects were screened for participation in the Self-Directed Biological Transformation Initiative (SBTI) study. After applying the eligibility criteria, informed consent was obtained from 119 participants with 65 subjects assigned to the Perfect Health (PH) Experimental group and 54 to the Relaxation/Vacation Control group. Age, gender, and BMI did not differ significantly between the two groups; however, it was subsequently determined that the baseline systolic blood pressure was lower in the PH group (Table 1). We tested for association between age, BMI (Kruskal-Wallis) and gender (Pearson chi-squared test). We used a non-parametric test (Kruskal-Wallis) to identify associations between continuous variables and treatment arm in order to guard against spurious results due to outliers. The *p*-values for association were 0.89, 0.33 and 0.58 respectively. These physiological variables were not associated with metabolite differences observed between the experimental and control groups and thus were not viewed as significant confounders in our study design. Data collected (yes/no) on caffeine usage (55% PH; 71% Control) was not statistically significant (*p* = 0.7) between groups (Pearson chi-squared test) and was lower in both groups than expected as it is estimated that 85% of individuals in the United States consume at least 1 caffeinated beverage per day^13^. Quantitative dosage information on caffeine usage was not available to enable rigorous assessment of potential confounding effects.

The hypothesis of the present study was that *Panchakarma* intervention (see Supplementary Methods S1) leads to distinct plasma metabolite changes compared to simple relaxation as a control. A broad spectrum of plasma metabolites representing 5 compound classes was assessed in fasting blood samples (baseline, day 0 and endpoint, day 6). Analytes were extracted from the plasma samples using the Biocrates Absolute*IDQ*^®^ p180 kit which includes all requisite calibration standards, internal standards, and Quality Control samples. Selective analyte detection is accomplished by use of a triple quadrupole tandem mass spectrometer (Waters Xevo^®^ TQ-S) operated in Multiple Reaction Monitoring (MRM) mode in which specific precursor to product ion transitions are measured for every analyte and stable isotope labeled internal standard. Two separate tandem mass spectrometric analyses are performed for each sample. For the analysis of acylcarnitines, glycerophospholipids, and sphingolipids, samples are introduced using a Flow Injection Analysis method (FIA-MS/MS). The analysis of amino acids and biogenic amines are performed by a UPLC (ultra-high pressure liquid chromatography, Waters Acquity) tandem MS method using a reversed phase analytical column for analyte separation (LC-MS/MS). The resulting chromatographic data were analyzed using Waters application TargetLynx™ and Biocrates Met*IDQ*™ software.

**Figure 1:**
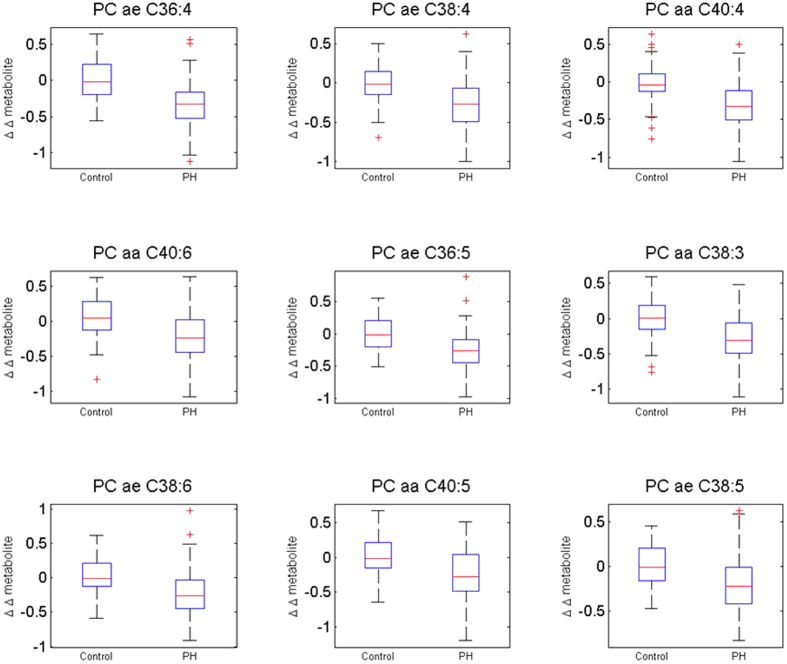
Box-and-Whisker plots of top 9 most differentially abundant plasma metabolites. Boxplots display change in log2 expression from baseline to post-intervention for the Control and Perfect Health (PH) groups. Delta was calculated by log2 transformation of metabolite expression data (μM), computing the difference between post-treatment and baseline for each patient, and computing the average of that difference separately for PH and control groups. Bars display standard deviation.

Analysis of the metabolomics data comparing pre- to post-intervention indicated that 12 metabolites significantly decreased in the PH compared to the control group after Bonferroni correction for multiple testing (adjusted *p* < 0.01) (*p*-values shown in Table 2; metabolite expression changes shown in Supplementary Table S2). Delta was calculated by log2 transformation of metabolite expression data (μM), computing the difference between post-treatment and baseline for each patient, and computing the average of that difference separately for PH and control groups. All 12 metabolites were classified as phosphatidylcholines; within this grouping, the phosphatidylcholine with the greatest decrease in abundance was PC ae C36:4, and the decrease in abundance between PH and control was moderate (delta = −0.34). The metabolites displaying the greatest differences between the PH and control groups are shown in Table 2; variation is displayed in Fig. 1. Within this set of metabolites, the trend of decreased metabolite expression was observed in 75–90% of the subjects (Table 3), indicating that a high frequency of subjects responded similarly to the PH treatment compared to control subjects. The changes in the top 10 most significant phosphatidylcholines separated the PH and control groups with an area under the curve (AUC) of 0.835.

**Figure 2:**
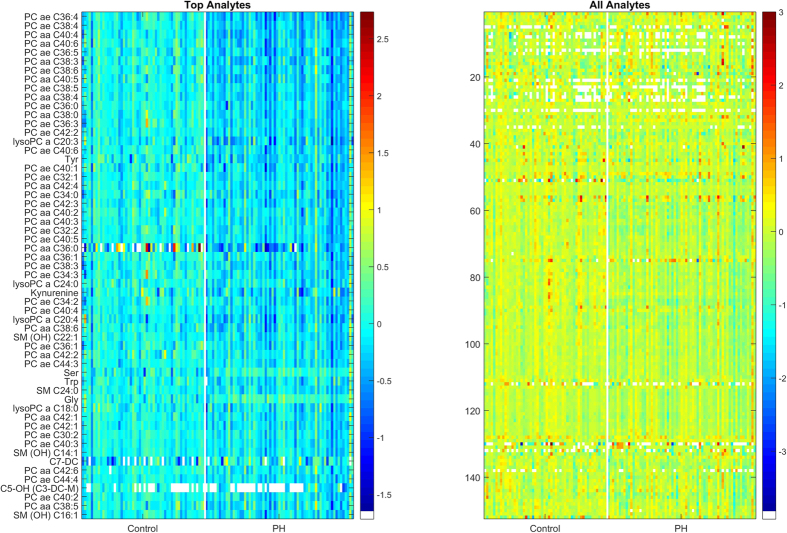
Hierarchical clustering of plasma metabolite changes. All plasma metabolites (left) and the metabolites with < 1% FDR (right) displayed as a heat map for Perfect Health (PH) and control groups.

Application of a less stringent FDR (10%) revealed an additional 57 metabolites that were differentially abundant between groups representing an expanded set of compound classes (see Table 4). The hierarchical clustering of metabolites is displayed in Fig. 2. In total, 70% of all differentially abundant features were classified as diacyl- or acyl-alkyl-phosphatidylcholines while the remaining features were annotated as lysophosphatidylcholines (9%), amino acids (9%), hydroxysphingomyelins (6%), acylcarnitines (4%), and biogenic amines (1.4%). PC aa C34:3 was the only phosphatidylcholine that increased in the PH compared to control. All 5 of the lysophosphatidylcholines detected were reduced in PH compared to control with the greatest decrease observed in 20:3. The 4 sphingolipids detected were also reduced in PH compared to control. Glutarylcarnitine (C5-DC) and hydroxyvalerylcarnitine (C5-OH) were reduced, and pimelylcarnitine (C7-DC) was increased in PH compared to controls. The biogenic amine kynurenine and amino acids tyrosine and tryptophan were reduced in the PH compared to controls. Glycine was modestly increased in PH compared to controls. Serine was increased in the PH compared to control group.

There were significant differences in baseline metabolite expression levels between study groups. However, baseline differences in expression were generally not associated with changes in expression observed after treatment. In regression analyses, controlling for baseline metabolite levels, the effect of treatment on change from baseline was significant for 18 metabolites; three of the 12 original metabolites dropped out (after Bonferroni correction) and 9 new differences were identified (Table 5). In particular, PH was associated with reductions in tyrosine (delta = −0.26, p = 0.000003), kyneurenine (delta = −0.29, p = 0.00005) and some lysophosphatildycholines (Table 5). The overall direction of the identified effects was similar regardless of whether baseline was adjusted or not (Supplementary Figures S3 and S4). We also examined gender effects by testing the metabolite expression change from baseline to study completion for each metabolite for association with experimental group controlling for gender using multiple regression with delta expression as the outcome. The minimum unadjusted *p*-value across tests (one test for each metabolite) is 0.07. There were no examples in which gender was found to be significant in this context.

Pathway overrepresentation analyses using previously described methods for those metabolites with known identities that differed at <1% FDR revealed that metabolites that differed between the PH and control groups may impact many different biochemical pathways (Table 6)^14^,^15^. Metabolites mapped onto 61 pathways derived from the KEGG, Reactome, SMPDB, EHMN, and Wikipathways databases. These include biochemical pathways (*p* < 0.05) involved in phospholipid synthesis, choline metabolism, opioid signaling, G-protein mediated events, acyl chain remodeling, HDL-mediated lipid transport, and lipoprotein metabolism.

## Discussion

The present study employed a targeted metabolomics approach to examine the effect of a controlled 6-day *Panchakarma*-based Ayurvedic retreat intervention on plasma metabolites in healthy human subjects. Highly significant reductions in 12 phosphatidylcholines and significant changes in an additional 57 metabolites classified as amino acids, biogenic amines, acylcarnitines, glycerophospholipids and sphingolipids were observed in the PH compared to the control group. Pathway overrepresentation analyses revealed that significant differentially abundant metabolites are associated with several different biochemical pathways. Significant pathways were largely related to signaling and lipid digestion, mobilization, transport and biosynthesis. Overall, 70% of all detected metabolites were classified as diacyl- or acyl-alkyl-phosphatidylcholines with the remaining metabolites annotated as lysophosphatidylcholines, amino acids, hydroxy-sphingomyelins, acylcarnitines, and biogenic amines. In addition, 93% of detected metabolites were reduced in abundance in the PH compared to control group. We speculate that the observed metabolite alterations may reflect the effect of one or more components of our intervention such as differences in diet between the experimental and control groups. Previous studies comparing dietary groups have reported distinct blood metabolite profiles associated with diet^16^,^17^. For example, lower serum phosphatidylcholine and sphingolipid levels have been reported in vegans and vegetarians compared to meat eaters^18^.

Phosphatidylcholines (PCs) are membrane phospholipids that consist of a glycerol core with a choline head group and two fatty acid residues. In the Western diet, eggs are a primary source of phospholipids, which exert broad effects on pathways related to inflammation and cholesterol metabolism^19^. Eggs and meat were not provided and only condiment amounts of dairy were given to the PH group during the study. Total plasma PC concentration has been reported as increased in obese men compared to controls^20^. Levels of PC 40.2 and 40.5, which were reduced in PH compared to control, have been reported as positively correlated with serum cholesterol in mice^21^. The acyl-alkyl-phosphatidylcholines C34:3, C40:6, and C44:4 were significantly decreased in PH compared to controls and interestingly have been reported as inversely related to type II diabetes risk^22^; although examining whether the profile differences are associated with disease risk is beyond the scope of this study.

Many dietary constituents serve as substrates that are metabolized by the gut microbiota into products that can alter host physiology. The significant alterations in plasma metabolites following PH are consistent with previous studies that reported that the gut microbiota and host metabolism are altered rapidly following a dietary shift^23^,^24^,^25^. The absence of meat and minimal dairy products in the PH diet are consistent with the alterations in plasma metabolites observed and may suggest that diet may have an impact on the plasma analytes reported here. The timeframe of these changes is consistent with reports comparing human subjects, using a cross over design, consuming an omnivorous or vegetarian diet. The gut microbiota composition displayed marked change after just 1 day with additional changes occurring over the following 6 days post-intervention^26^,^27^. Specialized species in the gut microbiota convert dietary choline and L-carnitine into trimethylamine (TMA) which is then converted into trimethylamine *N*-oxide (TMAO) upon oxidation in the liver^28^. Plasma and serum levels of the phosphatidylcholine metabolites choline, TMAO, and betaine are highly predictive of cardiovascular disease (CVD) risk^29^,^30^. Previous metabolomics studies have identified high levels of TMAO and acetylcarnitine following a meat diet^31^. The reduction of these substrates in the PH diet compared to control is consistent with reduced abundance of plasma phosphatidylcholine and acylcarnitine metabolites. The health benefits of the plant-based diets consumed by vegetarians and vegans are well described^32^,^33^.

Lysophosphatidylcholines (LPCs) are components of cell membranes and blood as well as some foods. They are derived from the partial hydrolysis of phosphatidylcholine, which removes one fatty acid; therefore, LPCs carry a single fatty acid. LPC can be metabolized into lysophosphatidic acid, which can act as a ligand for G-protein-coupled receptors that are linked to numerous disease states^34^,^35^. Epidemiological investigations have revealed associations between levels of specific LPCs and CVD risk that indicates a potential role of phospholipids in the development of CVD. Overall, all 5 of the LPCs detected were reduced in PH compared to control with the greatest decrease observed in 20:3. Probiotic supplementation experiments have reported similar reductions in fasting serum triglycerides and LPCs such as 16:1 and 20:3 compared to placebo controls^36^. Clinical studies on prehypertensive subjects, a known risk factor for atherosclerosis, reported higher levels of LPCs 16:1, 18:0, 20:4, C20:3 compared to controls^37^. Interestingly, these metabolites were observed as reduced in PH compared to controls. The vegetarian diet consumed by the PH group may, at least in part, improve lipid metabolism.

Sphingomyelins, are also membrane phospholipids with a ceramide core instead of glycerol and one polar head group, are synthesized through the transfer of phosphorylcholine from phosphatidylcholine to ceramide. In addition, sphingomyelins are components of lipoproteins, contained in the sheath surrounding some nerve cell axons, and are involved in signal transduction through nuclear factor-κB pathways. Sphingolipids also occur naturally in foods with the highest levels found in dairy and soybeans followed by meat^38^. Sphingomyelins, a major phospholipid species contained in eggs, may have a regulatory role in cholesterol absorption and inflammation^19^. The 4 sphingolipids detected were reduced in PH compared to control subjects and may reflect the reduction of foods with high sphingolipid content such as cream, cheese, and eggs in the PH group.

To date, very few controlled studies on *Panchakarma* have been undertaken and most have occurred in the past decade. To our knowledge, this represents the first study to examine the effect of a *Panchakarma*-based Ayurvedic intervention on plasma metabolites in a controlled clinical trial. The present study applied a targeted and standardized metabolomics platform. However, there are limitations of the study that include the moderate cohort size as well as the multiple components inherent to this specific type of Ayurvedic intervention. Thus, while the change in diet offers the simplest potential explanation of the findings, we cannot overlook the possible impact of herbs with potential hypolipidemic effects, yoga, oils and prebiotic fiber on the PH group plasma profile changes^7^,^8^,^9^,^10^. However, at the fairly low dosages used (see Supplementary Methods S1) and given observations that herbs exert detectable therapeutic effects more slowly compared to dietary changes, we speculate that diet produced a greater impact compared to herbs on the observed plasma metabolite changes; future studies are necessary to confirm this hypothesis. While caffeine usage was lower than the national average for both groups and not statistically significant between groups, quantitative dosage information on caffeine usage was not collected to enable more rigorous assessment of potential confounding effects. Future studies could include the collection of caffeine dosage information and test for associated confounding effects. One of the challenges inherent to our study was establishing physiologically significant alterations in a relatively short study period. While the absolute magnitude of metabolite changes is relatively small, the significance is reinforced by the coherence and directionality of the pathways they highlight. Therefore, due to relatively small sample sizes, modest effect sizes of metabolite changes, and short duration of the intervention, our findings should be interpreted as hypothesis-generating.

While numerous studies have examined the health benefits of integrative medicine practices such as meditation, yoga, herbal supplements and Ayurvedic diet, few studies have undertaken a systems biology approach to examine the effects of such practices on pathways and functional networks in multiple tissues. Thus, the forthcoming integration of the metabolomics data with genomics, metatranscriptomics, and other clinical data from the SBTI study in a systems biology analysis may yield additional insights. Subsequent studies may include expanded cohorts and a global, untargeted metabolomics approach for additional observational power and insight. Future studies may focus on single variables such as diet, meditation or herbs alone to dissect the specific molecular features and biological mechanisms related to the various components of this Ayurvedic intervention. Future directions include clinical trials that apply *Panchakarma*-based Ayurvedic treatment to clinical populations such as those with cardiometabolic disease, cancer, and neuro-psychiatric disease. In conclusion, the observed plasma metabolite alterations may reflect a *Panchakarma*-induced modulation of metabotypes. The *Panchakarma*-based Ayurvedic intervention promoted statistically significant changes in plasma levels of phosphatidylcholines, sphingomyelins and others in only 6 days.

## Methods

### Study Design

The Self-Directed Biological Transformation Initiative (SBTI) was designed to study the effects of a 6-day Ayurvedic retreat (Perfect Health program) on biological markers of cell biology, genome, metabolome and the microbiome as well as on psychological indices of well-being. This clinical trial compared the effects of participation in a *Panchakarma*-based Ayurvedic retreat called the Perfect Health Program (PH) at the Chopra Center for Wellbeing at the La Costa Resort in Carlsbad, CA to simple relaxation (i.e. a resort vacation control group) at the La Costa Resort in Carlsbad, CA. Based on principles of *Panchakarma*, Ayurvedic Medicine’s principle cleansing and rejuvenation protocols, PH incorporates various treatment modalities such as a vegetarian diet, Ayurvedic herbs using the Zrii Purify™ herbal program per manufacturer’s instructions, meditation, yoga, specialized Ayurvedic oil massage, heat therapies, and lectures on self-care and well-being (see Supplementary Methods S1 for detailed herb, oil, and prebiotic fiber cleanse protocol). The PH program employs a palliation approach, which combines gentle reduction and tonification therapy, and is focused on *Purvakarma* (preparatory techniques of *Panchakarma*) procedures and 2 of the 5 main *Pradhankarma* techniques of elimination, namely *Virechana* and *Nasya*. Gentle *Virechana* is accomplished using the purgative herbs of the Zrii Purify™ herbal program. All study participants were housed onsite at the resort. The study was approved by the University of California, San Diego Human Subjects Research Protections Program Investigational Review Board and performed in accordance with institutional guidelines for research with human subjects. The SBTI study was registered at ClinicalTrials.gov as NCT02481544.

#### Recruitment and Study Participants

Women and men were recruited from the greater San Diego and Los Angeles areas; however, a few participants came from greater distances. Those traveling outside of Pacific Standard Time had to be acclimated to the current time zone by the first day of study using the one hour per day criteria. Recruitment was via email announcements on the Chopra Center and UC San Diego faculty and staff email list serves. Study recruitment flyers stated that the 6-day SBTI study would be conducted at the Chopra Center for Wellbeing located at La Costa Resort to learn more about the effects of the Ayurvedic program on key biochemical, physiological, and psychosocial endpoints, as compared to individuals who will not take the program.

Eligible participants were healthy English-speaking (given the lecture component) women and men aged 30–80 years with no current major medical conditions including cancer, heart disease, autoimmune disease or diabetes. Participants were not smokers, pregnant, taking antidepressants or hormone replacement therapy, or have a current diagnosis of PTSD. Participants were willing to refrain from drinking more than one alcoholic beverage per day during the weeklong stay at La Costa Resort. Other study exclusion criteria included a Body Mass Index (BMI) of 35 kg/m^2^ or greater; individuals who had ever previously participated in the PH, Balancing the Body program, other Chopra Center programs, or a yoga or meditation retreat of any kind within the past 12 months; self-reported use of illicit drugs, including marijuana; and those taking prescription medication not prescribed by their physician.

Potential participants were screened for eligibility, learned details of study involvement, and were asked to consider their commitment and availability before they were enrolled into the study. It was emphasized that this included a commitment to stay in the study even if they were not randomized into their preferred group. Informed consent was obtained from all the subjects. Those electing to participate in the SBTI study were then assigned into the PH group or the Resort group and invited to participate in one of six different study cohorts. The treatment group assignments did not follow a strict randomization; only ~80% of subjects were randomized.

#### Study Interventions

The intervention groups were not blinded and site investigators and study personnel knew the group to which group participants had been randomized. The study was designed to minimize contact between the two groups or knowledge about the daily schedule of the other group. Upon arrival to the resort, participants were given a one-hour orientation meeting with the study team where they learned about the overall study schedule, the assessment schedules and procedures of the study.

##### Perfect Health (PH) Experimental Group

The PH Program, which has been offered at the Chopra Center for Wellbeing for approximately 15 years, is a *Panchakarma*-based immersive retreat of detoxification and rejuvenation based on core principles from the Ayurvedic system of medicine. The PH program addresses physical, emotional and spiritual wellbeing through daily practices such as Ayurvedic diet, herbs, yoga, meditation, specialized massage and educational lectures on diet and self-care. Key components of the program include physical cleansing through ingestion of Ayurvedic herbs, prebiotic fiber and oils that support the body’s natural detoxification pathways and facilitate healthy elimination, two Ayurvedic meals daily (breakfast and lunch) which provided a light plant-based diet, daily Ayurvedic massage treatments, and heat therapy through the use of sauna and/or steam. The PH study group also participated in two daily group meditations and two daily *Hatha* yoga practices. During the program, participants received a one-hour integrative medical consultation by a physician and a follow up with an Ayurvedic Health Educator. The teachers of the PH Program delivered their standard program to the SBTI participants.

##### Control (Resort Vacation) Group

Participants in the Resort Relaxation control group were advised to do what they would normally do on a vacation with the following restrictions: they were asked not to engage in more exercise than they would in their normal lifestyle and to refrain from using Chopra Center spa Ayurvedic services. Resort participants were also asked not to drink ginger tea or take gingko biloba during the two days prior to and during the study week.

### Assessments

All study participants underwent an identical set of assessments, which included completing study questionnaires, saliva sampling, stool collection and heart rate monitoring using the “MultiSense™ Memory device at baseline upon arrival to the resort and on day 6 of the PH or Resort Vacation (Control) programs. In addition, three seated blood pressures, height, weight, waist and hip circumference and a fasting blood sample were collected upon arrival to the resort and the day completing the PH or Resort programs. Fasting blood samples were collected between 6:00 AM and 10:00 AM at baseline and on the final day (day 6) of the intervention for downstream metabolomics analysis. Morning fasting blood samples were obtained by IV catheter in purple top Vacutainers (Becton Dickinson) and immediately placed on ice. Samples were centrifuged at 4 °C and the plasma immediately stored at −80 °C until the assay was performed. Frozen plasma samples were shipped to the Duke Metabolomics Shared Resource on dry ice. Non freeze-thawed samples were used for analysis.

#### Metabolomics

All metabolomic analyses were done blind to treatment arm. Fasting EDTA plasma samples (baseline and endpoint) from SBTI participants were analyzed using the Absolute*IDQ*^®^ p180 kit (Biocrates LifeSciences AG, Innsbruck, Austria). The use of this commercial kit and its associated software tool (MetIDQ™) along with a state-of-the-art LC-MS/MS system (Acquity UPLC (Waters) and Xevo^®^ TQ-S triple quadrupole (Waters) mass spectrometer) provided highly reproducible and sensitive analyses. There are 186 metabolites targeted with this kit, representing five classes of metabolites. LC-MS/MS was used for amino acids and biogenic amines analyses. FIA-ESI-MS/MS was used for acylcarnitines, phospholipids, sphingolipids and hexoses. Multiple reaction monitoring (MRM) mode was employed for all analyses, providing high specificity and sensitivity.

Sample preparation, LC-MS/MS analysis and FIA-MS/MS analysis was performed in the Duke Proteomics and Metabolomics Shared Resource according to the Biocrates p180 Kit User Manual, which contains a detailed Standard Operating Procedure which Biocrates validated in their labs in Austria. Scientists in the Duke Proteomics and Metabolomics Shared Resource received hands-on training from Biocrates scientists to ensure the SOP is correctly used for all analyses. The Biocrates p180 kit includes internal standards, three quality control samples for LC-MS/MS and FIA-MS/MS analysis, and calibration standards for LC-MS/MS analyses. Using the AbsoluteIDQ p180 kit, the LC-MS/MS analyses are fully quantitative for 22 amino acids and 18 biogenic amines, using 20 stable-isotope labeled internal standards with 62 retention time scheduled transitions. For the LC-MS/MS analyses, the kit includes a full 7-point calibration curve (run in duplicate; start and end of analysis cohort) and QC samples at low, medium and high concentrations (each run in triplicate on each of the three plates used in this study). The FIA-MS/MS analyses are semi-quantitative for 40 Acylcarnitines, 15 Sphingomyelins, 90 Phosphatidylcholines (PC) and Lyso-PCs. Analyte-specific MS/MS transitions were monitored for 156 analytes and 11 stable-isotope internal standards. For these FIA-MS/MS analyses, a single point calibration curve is used, along the triplicate analyses of the three QC standards on each plate. The QC samples in the kit are comprised of 57 metabolites, covering amino acids, biogenic amines, glycerophospholipids and acylcarnitines, with the concentration of the analytes in the three QC samples corresponding to low, mid and high levels relative to the quantitation range for each of these metabolites. Metabolites were extracted from human plasma using the Biocrates 96-well plate system for protein removal, internal standard normalization and derivatization.

All data analysis is performed via the Biocrates MetIDQ software, which uses the MetLIMS module to register plates, blanks, standards, QC samples, study samples and projects. The UPLC-MS/MS data were imported into Waters application TargetLynx™ for peak integration, calibration, and concentration calculations. The UPLC-MS/MS data from TargetLynx™ and FIA-MS/MS data were analyzed using Biocrates Me*tIDQ*™ software. The MetVal module is used to process the MS/MS data – blanks, calibration curves, QC samples and samples - using standard methods, and the results are presented in multiple graphical formats, providing a comprehensive overview of the accuracy and data quality. The MetStat module in MetIDQ is used to summarize the assay results, allowing the sample measurement values to be sorted by analyte class and combined with the validation results (QC samples), with the final data results stored in an Oracle database. All metabolite values are reported in micromolar units.

The metabolite nomenclature has been published previously^39^. Briefly, the lipid side chain composition was denoted “Cx:y” where x and y refer to the number of carbon atoms and double bonds, respectively. Acylcarnitines, glycerophospholipids and sphingolipids were abbreviated according to the fatty acid side chain. All glycerophospholipids in the panel were phosphatidylcholines. Phosphatidylcholines with one fatty acid side chain bound with an acyl bond are labelled as “LysoPC”, “PC aa” indicates 2 acyl side chains, and “PC ae” indicates one acyl and one alkyl side chain. Amino acids were labelled with standard abbreviations.

##### Plasma metabolite concentrations

All samples were randomized and submitted for analyses in a blinded manner. Plasma metabolite concentrations were determined with the AbsoluteIDQ^®^ p180 kit (Biocrates, Innsbruck, Austria) as described above. A 10 μL aliquot of the mixture of internal standards was added to the appropriate wells of the 96-well extraction plate, followed by the addition of 10 μL of each plasma sample. The wells of the plate were dried under a gentle stream of nitrogen (SPE Dry, Jones Chromatography, Lakewood, CO). The samples were derivatized with phenyl isothiocyanate (Sigma, St. Louis, MO), then eluted with 5 mM ammonium acetate (JT Baker, Phillipsburg, PA) in methanol (LC-MS Grade, EMD Millipore, Darmstead, Germany). Samples were diluted with either 40% methanol in water (Optima LC/MS Grade, Fisher Chemical, Fair Lawn, NJ) for UPLC analysis (10:1) or running solvent (a proprietary mixture provided by Biocrates) for flow injection analysis (20:1).

The LC separation of amino acids and biogenic amines was performed using a Waters (Milford, MA) Acquity UPLC with a Waters Acquity 2.1 mm x 50 mm 1.7 μm BEH C18 column fitted with a Waters Acquity BEH C18 1.7 μm Vanguard guard column. Acylcarnitines, sphingolipids, and glycerophospholipids, were analyzed by flow injection. Samples for both UPLC and flow injection analysis were introduced directly into a Xevo TQ-S mass spectrometer (Waters) using electrospray ionization (ESI) operating in the MRM mode. MRM transitions (compound-specific precursor to product ion transitions) for each analyte and internal standard were collected over the appropriate retention time. The UPLC data were imported into Waters application TargetLynx™ (Waters) for peak integration, calibration, and concentration calculations. The UPLC data from TargetLynx™ and flow injection data were analyzed using Biocrates MetIDQ™ software.

The p180 kit includes LC-MS calibration standards at seven concentrations as well as QC samples at three different concentrations. A full set of calibration standards were run before and after sample analyses, and each of the three levels of Biocrates supplied QC samples were analyzed in triplicate from each plate. These results show excellent precision (see Supplementary Table S5. In addition to the Biocrates QC samples, a study pool QC sample (SPQC) of equal volumes of the first 80 samples was created for analysis as a SPQC standard on each plate. This sample was frozen in aliquots of an appropriate volume to be analyzed with all three of the plates analyzed in this study. The pooled sample was prepared in the same way as the study samples. An aliquot of the pooled sample was extracted with each sample plate and was analyzed in triplicate. This sample was injected once before, once during, and once after the samples on each plate in order to measure the performance of the assay during sample analysis. The SPQC samples also show excellent reproducibility (Table 7), with precision ranging from a high of 11.7% CV (biogenic amines) to a low of 4.61% CV (sphingolipids).

##### Statistical methods

The main hypothesis was that PH leads to metabolic changes that are distinct from that in the Control group. Relationships between demographics and treatment arm were tested using a non-parametric test for continuous variables and Chi-squared test for categorical variables. Metabolites with greater than 50% missing values were dropped and expression levels were log_2_ transformed. Differences in log_2_ transformed metabolite levels were calculated between baseline and 6 days after treatment. Independent *t*-test for each metabolite was used to assess the relationship between the computed change from baseline to endpoint in the PH versus Control groups. This analysis did not co-vary for baseline metabolite levels. We also ran a multiple regression analyses comparing the change from baseline in metabolites between treatment groups after controlling for baseline metabolite expression (in micromolar units). Bonferroni correction (adjusted *p* < 0.01) was employed for primary analyses as it was more stringent. Secondary analyses were performed using a lower threshold of Benjamini-Hochberg FDR at 10% for hypotheses generation. Preliminary biochemical pathway analyses were conducted using the open-source IMPaLA integrated pathway analysis tool^14^ by selecting metabolites that were significant at 1% FDR and whose chemical identification was known. Pathway overrepresentation analysis was performed with IMPaLA and biochemical pathways that were mapped with *p*-values < 0.05 were reported. Q-values indicate the FDR which results from correcting the *p*-values for multiple testing using a method previously described by Benjamini and Hochberg^40^. Principal Components Analysis (PCA) was performed to summarize overall data trends (see Supplementary Figure S6). Box plots depict the data.

## Additional Information

**How to cite this article**: Peterson, C. T. *et al*. Identification of Altered Metabolomic Profiles Following a *Panchakarma*-based Ayurvedic Intervention in Healthy Subjects: The Self-Directed Biological Transformation Initiative (SBTI). *Sci. Rep.* **6**, 32609; doi: 10.1038/srep32609 (2016).

## Supplementary Material

Supplementary Information

## Figures and Tables

**Table 1:** Baseline demographics of Perfect Health and control subjects.

mean ± SD	Perfect Health	Control
N	54	65
Age (years)	54.7 (11.9)	54.2 (11.8)
Gender (% female)	81%	77%
BMI (kg/m^2^)	24.6 (4.9)	23.8 (4.1)
Systolic BP (mmHg)*	120 (18)	113 (14)
Diastolic BP (mmHg)	77 (13)	74.5 (11)
Heart rate (bpm)	68.1 (11.6)	68.1 (8.3)
Alcohol use (%)	61%	58%
Caffeine use (%)	55%	71%

General subject characteristics at time 0. Starred items have *p* < 0.01.

**Table 2:** Top 12 most differentially abundant plasma metabolites (Bonferroni adjusted *p* < 0.01).

Metabolite	∆PH-∆Control	*p*-value	FDR
PC ae C36:4	−0.340	1.97E-08	3.00E-06
PC aa C40:4	−0.263	4.00E-06	2.20E-04
PC ae C38:4	−0.246	4.00E-06	2.20E-04
PC aa C40:6	−0.278	7.00E-06	2.29E-04
PC ae C36:5	−0.265	8.00E-06	2.29E-04
PC aa C38:3	−0.262	2.00E-05	4.75E-04
PC ae C38:6	−0.254	2.20E-05	4.75E-04
PC aa C40:5	−0.261	3.60E-05	6.91E-04
PC ae C38:5	−0.204	4.50E-05	7.62E-04
PC aa C38:4	−0.205	5.00E-05	7.62E-04
PC ae C36:0	−0.211	5.80E-05	7.79E-04
PC aa C38:0	−0.215	6.10E-05	7.79E-04

Delta was calculated after log2 transformation of metabolite expression data (μM), computing the difference between post-treatment and baseline for each patient, and computing the average of that difference separately for PH and control groups.

**Table 3:** Fraction of subjects with decreased metabolite expression.

Metabolite	Control	PH
PC ae C36:4	0.54	0.89
PC ae C38:4	0.52	0.82
PC aa C40:4	0.52	0.83
PC aa C40:6	0.39	0.74
PC ae C36:5	0.52	0.85
PC aa C38:3	0.48	0.79
PC ae C38:6	0.52	0.80
PC aa C40:5	0.52	0.74
PC ae C38:5	0.50	0.75
PC aa C38:4	0.48	0.80
PC ae C36:0	0.41	0.74
PC aa C38:0	0.48	0.80

**Table 4:** Additional differentially abundant plasma metabolites (FDR < 10%).

Target	∆PH-∆Control	p-value	FDR
PC ae C36:3	−0.22	9.90E-05	1.15E-03
PC ae C42:2	−0.19	1.82E-04	1.86E-03
lysoPC a C20:3	−0.26	1.90E-04	1.86E-03
PC ae C40:6	−0.20	1.95E-04	1.86E-03
Tyr	−0.23	2.13E-04	1.91E-03
PC ae C40:1	−0.25	2.67E-04	2.25E-03
PC ae C32:1	−0.16	4.14E-04	3.31E-03
PC aa C42:4	−0.18	4.57E-04	3.47E-03
PC ae C34:0	−0.21	6.17E-04	4.47E-03
PC ae C42:3	−0.19	6.92E-04	4.78E-03
PC aa C40:2	−0.19	7.83E-04	5.16E-03
PC aa C40:3	−0.16	8.15E-04	5.16E-03
PC ae C32:2	−0.17	8.60E-04	5.23E-03
PC ae C40:5	−0.16	9.25E-04	5.36E-03
PC aa C36:0	−0.48	9.52E-04	5.36E-03
PC aa C36:1	−0.19	1.04E-03	5.66E-03
PC ae C38:3	−0.18	1.17E-03	6.11E-03
PC ae C34:3	−0.19	1.28E-03	6.40E-03
lysoPC a C24:0	−0.18	1.31E-03	6.40E-03
Kynurenine	−0.23	2.00E-03	9.52E-03
PC ae C34:2	−0.17	2.39E-03	1.10E-02
PC ae C40:4	−0.14	2.60E-03	1.16E-02
lysoPC a C20:4	−0.21	2.80E-03	1.22E-02
PC aa C38:6	−0.18	3.08E-03	1.30E-02
SM (OH) C22:1	−0.14	3.20E-03	1.31E-02
PC ae C36:1	−0.16	3.31E-03	1.32E-02
PC aa C42:2	−0.16	3.48E-03	1.33E-02
PC ae C44:3	−0.13	3.50E-03	1.33E-02
Ser	0.16	3.58E-03	1.33E-02
Trp	−0.17	5.07E-03	1.84E-02
SM C24:0	−0.13	5.22E-03	1.85E-02
Gly	0.14	5.48E-03	1.89E-02
lysoPC a C18:0	−0.17	6.95E-03	2.35E-02
PC aa C42:1	−0.13	7.27E-03	2.40E-02
PC ae C42:1	−0.13	7.72E-03	2.45E-02
PC ae C30:2	−0.13	7.72E-03	2.45E-02
PC ae C40:3	−0.12	8.28E-03	2.57E-02
SM (OH) C14:1	−0.12	8.60E-03	2.61E-02
C7-DC	0.22	1.07E-02	3.19E-02
PC aa C42:6	−0.12	1.35E-02	3.93E-02
PC ae C44:4	−0.11	1.38E-02	3.93E-02
C5-OH (C3-DC-M)	−0.18	1.39E-02	3.93E-02
PC ae C40:2	−0.12	1.68E-02	4.63E-02
PC aa C38:5	−0.16	1.76E-02	4.77E-02
SM (OH) C16:1	−0.12	1.83E-02	4.89E-02
PC aa C36:4	−0.08	2.05E-02	5.36E-02
PC ae C38:0	−0.19	2.16E-02	5.56E-02
PC aa C32:0	−0.11	2.54E-02	6.44E-02
Phe	−0.10	2.64E-02	6.57E-02
C5-DC (C6-OH)	−0.18	2.78E-02	6.83E-02
PC aa C34:3	0.16	3.15E-02	7.61E-02
Orn	0.13	3.24E-02	7.70E-02
PC ae C34:1	−0.11	3.37E-02	7.88E-02
PC ae C42:4	−0.10	3.72E-02	8.57E-02
lysoPC a C26:0	−0.14	3.81E-02	8.65E-02
lysoPC a C16:1	−0.17	3.87E-02	8.66E-02
PC aa C32:1	−0.22	4.07E-02	8.97E-02

Delta was calculated after log2 transformation of metabolite expression data (μM), computing the difference between post-treatment and baseline for each patient, and computing the average of that difference separately for PH and control groups.

**Table 5:** Metabolites showing a significant treatment effect after Bonferroni correction before and after controlling for baseline metabolite levels.

Baseline Unadjusted	Controlling for Baseline level
PC aa C38:0	
	Kynurenine
	PC aa C36:1
PC aa C38:3	PC aa C38:3
PC aa C38:4	PC aa C38:4
PC aa C40:4	PC aa C40:4
PC aa C40:5	PC aa C40:5
PC aa C40:6	PC aa C40:6
	PC ae C34:0
	PC ae C34:2
PC ae C36:0	PC ae C36:0
	PC ae C36:3
PC ae C36:4	PC ae C36:4
PC ae C36:5	PC ae C36:5
	PC ae C38:3
PC ae C38:4	PC ae C38:4
PC ae C38:5	
PC ae C38:6	
	Tyr
	lysoPC a C20:3
	
	lysoPC a C24:0

The first column shows the 12 metabolites whose change from baseline to end point differed significantly between PH and Control groups after Bonferroni correction for multiple testing (adjusted p < 0.01). The second column shows the 18 metabolites that differed between treatment groups in a multiple regression model controlling for baseline metabolite levels (Bonferroni adjusted p < 0.01). The overall direction of significance and changes remained the same regardless of whether baseline was controlled or not (See S5 and S6).

**Table 6:** Pathway mapping of differentially abundant plasma metabolites (*p* < 0.005).

Pathway Name	Pathway DB	*p*-value
Acyl chain remodeling of CL	Reactome	0.0049
Acyl chain remodelling of PC	Reactome	0.0049
Ca-dependent events	Reactome	0.0049
Choline metabolism in cancer - Homo sapiens (human)	KEGG	0.0049
Fcgamma receptor (FCGR) dependent phagocytosis	Wikipathways	0.0049
Glycerophospholipid metabolism - Homo sapiens (human)	KEGG	0.0049
G-protein mediated events	Reactome	0.0049
HDL-mediated lipid transport	Reactome	0.0049
Linoleate metabolism	EHMN	0.0049
Lipid digestion, mobilization, and transport	Wikipathways	0.0049
Lipid digestion, mobilization, and transport	Reactome	0.0049
Lipoprotein metabolism	Reactome	0.0049
Opioid Signalling	Reactome	0.0049
Opioid Signalling	Wikipathways	0.0049
Phospholipid Biosynthesis	SMPDB	0.0049
phospho-PLA2 pathway	Reactome	0.0049
PLC beta mediated events	Reactome	0.0049

Pathway overrepresention for those metabolites with known identities that differed at < 1% FDR.

**Table 7:** Precision of the P180 analyses as assessed via the nine analyses of the study pool QC sample.

Analyte Class	Average % CV
Amino Acids	10.4
Biogenic Amines	11.7
Glycerophospholipids	5.89
Sphingolipids	4.61
Acylcarnitines	8.05

This sample was analyzed in triplicate on each of the three plates used for the cohort analyses.
