# Corrigendum: Identification of Altered Metabolomic Profiles Following a *Panchakarma*-based Ayurvedic Intervention in Healthy Subjects: The Self-Directed Biological Transformation Initiative (SBTI)

**DOI:** 10.1038/srep34678

**Published:** 2016-10-10

**Authors:** Christine Tara Peterson, Joseph Lucas, Lisa St. John-Williams, J. Will Thompson, M. Arthur Moseley, Sheila Patel, Scott N. Peterson, Valencia Porter, Eric E. Schadt, Paul J. Mills, Rudolph E. Tanzi, P. Murali Doraiswamy, Deepak Chopra

Scientific Reports 6: Article number: 32609; 10.1038/srep32609 published online: 09 09 2016; updated: 10 10 2016.

In this Article additional disclosures should have been included in the Competing Financial Interests statement. The corrected statement is included below. The Authors apologize for these omissions.

The Competing financial interests statement in this Article should read:

“S.P. and V.P. are employed by the Chopra Center. D.C. is a co-founder and a co-owner of the Chopra Center. The Chopra Center offers the Perfect Health program commercially; however, none of the study subjects were charged for the participation in the program.”

