# Supplementary material for: Identification of Altered Metabolomic Profiles Following a Panchakarma-based Ayurvedic Intervention in Healthy Subjects: The Self-Directed Biological Transformation Initiative (SBTI)
Source: Sci Rep. 2016 Sep 9;6:32609. doi: 10.1038/srep32609 (PMC5017211; doi:10.1038/srep32609)
Supplement: Supplementary Information [file srep32609-s1.doc]

**Identification of Altered Metabolomic Profiles Following a *Panchakarma*-based Ayurvedic Intervention in Healthy Subjects: The Self-Directed Biological Transformation Initiative (SBTI)**

**Christine Tara Peterson**^1,2*^**, Joseph Lucas**^3^**, Lisa St. John-Williams**^4^**, J. Will Thompson**^4^**, M. Arthur Moseley**^4^**, Sheila Patel**^5,6^**, Scott N. Peterson**^7^**, Valencia Porter**^5,6^**, Eric E. Schadt**^8^**,****Paul J. Mills**^1^**, Rudolph E. Tanzi**^9^**, P. Murali Doraiswamy**^10^ **and Deepak Chopra**^2,5,6^

^1^Center of Excellence for Research and Training in Integrative Health, Department of Family Medicine and Public Health, University of California San Diego, La Jolla, California, USA

^2^Department of Ayurveda and Yoga Research, Chopra Foundation, Carlsbad, California, USA

^3^Proteomics Core Facility, Duke University, Durham, North Carolina, USA

^4^Proteomics and Metabolomics Shared Resource Center for Genomic and Computational Biology, Duke University Medical Center, Durham, North Carolina, USA

^5^Chopra Center for Wellbeing, Carlsbad, California, USA

^6^Department of Family and Preventive Medicine, University of California San Diego, La Jolla, California, USA

^7^Infectious & Inflammatory Disease Center, Sanford Burnham Prebys Medical Discovery Institute, La Jolla, California, USA

^8^Icahn School of Medicine at Mount Sinai, New York, New York, USA

^9^Genetics and Aging Research Unit, Department of Neurology, Massachusetts General Hospital and Harvard Medical School, Boston, Massachusetts, USA

^10^ Departments of Psychiatry and Medicine and the Duke Institute for Brain Sciences, Behavioral Sciences, Duke University Health System, Durham, North Carolina, USA

***** chpeterson@ucsd.edu

**S1. Supplementary Methods**

Program Overview (for Experimental Group):

1. Nutritional Guidelines for 7-day Wash Out Period Prior to PH Intervention
   1. All food should be freshly prepared, nutritious, and appetizing; canned foods and leftovers should be minimized.
   2. Eat light foods that are easy to digest, such as rice, soups, and lentils.
   3. Favor freshly steamed or very lightly sautéed vegetables.
   4. Avoid fried foods.
   5. Avoid ice-cold foods and drinks.
   6. Reduce or eliminate your intake of dairy products.
   7. Avoid fermented foods and drinks. This includes vinegar, pickled condiments, and cheeses.
   8. Avoid alcohol, marijuana, and other recreational chemicals.
   9. Keep oils to a minimum.
   10. Favor lighter grains such as barley or basmati rice.
   11. Minimize refined sugars; small amounts of honey may be used, but do not cook with it.
   12. Most nuts, which are oily, heavy, and salted, should be avoided. However, unsalted sunflower, pumpkin, flax, or sesame seeds may be eaten.
   13. If you cannot entirely eliminate animal products, favor the white meat of turkey or chicken. Avoid red meats, particularly pork and beef.
   14. Drink hot water with sliced fresh ginger frequently throughout the day.
   15. Do not eat until you are definitely hungry and do not overeat.
   16. Do not eat until the prior meal has been fully digested (three to six hours).
2. Ayurvedic Herbal Cleanse (Zrii Purify™) Ingredients (generally regarded as safe designation) (6 days)
3. AM Detox Herbal Capsules (per serving)
   1. Potassium glycinate (40 mg)
   2. Ayurveda Herbal Detox Blend (1,350 mg): Manjishtha (*Rubia cordifolia*) root, Guduchi (*Tinospora cordifolia*) root, Kutki (*Picrorhiza kurroa*) root, Punarnava (*Boerhavia diffusa*) root, Neem (*Azadirachta indica*) leaf, Musta (*Cyperus* *rotundus*) root, Gokshura (*Tribulus* *terristris*) aerial parts, Bhumyamalaki (*Phyllanthus* *niruri*) whole plant
   3. Herbal Detox Blend (280 mg): Dandelion root, Burdock root, Milk Thistle seed, Artichoke leaf, Parsley leaf, Mullein leaf, Uva Ursi leaf
   4. Nutraceutical Detox Blend (300 mg): Alpha-lipoic acid, calcium D-glucarate, N-acetyl cysteine, inositol
4. PM Cleanse Ayurvedic Herbal Capsules (per serving)
   1. Magnesium oxide (334 mg)
   2. Ayurveda Herbal Cleanse Blend (860 mg): *Cascara Sagrada* bark, Chitrak (Plumbago *zeylanica*) root, Marshmallow root, Slippery Elm bark, Prune fruit, Fennel seed, Peppermint leaf, Ginger root
5. Detox Oil (per serving)
   1. Vitamin D3 (50 IU)
   2. Vitamin E (d-alpha tocopherol acetate) (35 IU)
   3. Detox Blend (15 mL): Sesame oil, Flaxseed oil, Olive oil, Hempseed oil, Evening Primrose oil, Borage oil
6. Fiber-Herb Blend (per serving)
   1. Fiber Blend (13 g): Psyllium husk, Rice bran, Cellulose (plant-derived), Oat fiber, Inulin, Guar Gum, Gum Acacia, Flax seed, Orange peel
   2. Ayurvedic Herbal Blend (10.5 g): Triphala (*Emblica officinalis, Terminalia chebula, Terminalia belerica* fruit), Aloe Vera gel leaf extract (*Aloe barbadensis),* Slippery Elm bark, Buckthorn bark, Fennel seed, Fenugreek seed
7. Daily PH Cleanse Routine and Diet (6 days)
   1. Upon Rising: Take 4 AM Detox Capsules with 8 oz. of water at least 15 minutes prior to breakfast and at least 15 minutes before taking the Fiber-Herb Blend.
   2. Breakfast: Take 1 scoop of Fiber Blend with 8 oz. of water, 1 Tbsp. Detox Oil, and a sensible meal
   3. Freshly prepared Ayurvedic breakfast meal provided.
   4. Lunch: Ayurvedic meal provided.
   5. Dinner: Take 1 scoop of Fiber Blend with 8 oz. of water, 1 Tbsp. Detox Oil, and Ayurvedic meal on your own.
   6. Bedtime: Take 2 capsules PM Cleanse with 8 oz. of water before going to bed and at least 15 minutes after taking Fiber Blend.
   7. Drinks
      1. Plenty of water every day – about six 8-oz. glasses.
      2. Hot water with sliced fresh ginger frequently throughout the day.
      3. Herbal, non-caffeinated teas.
      4. Vegetable juice.
      5. Avoid alcohol, soda and caffeine.
   8. Yoga: Two 1-hour yoga classes per day.
   9. Meditation: Two to three 20-minute mantra-based silent meditation practices per day.
   10. Massage: Hour-long Ayurvedic massage treatment and external oleation per day.
   11. Heat therapies: Choice of dry or wet sauna and *Ushma Svedana* (steam tent) treatment.
   12. Lectures: Practical morning and afternoon lectures on well-being, food and self-care.

**S2. Delta effect size for metabolite expression (∆PH-∆Control)**

| **Target** | **Delta** | **p-value** | **FDR** |
| --- | --- | --- | --- |
| Ala | -0.04584 | 0.451382 | 0.536016 |
| Arg | -0.14295 | 0.133978 | 0.218975 |
| Asn | 0.030164 | 0.487255 | 0.565365 |
| Asp | 0.177743 | 0.173972 | 0.26182 |
| Cit | -0.06874 | 0.189139 | 0.276434 |
| Gln | 0.062642 | 0.142534 | 0.228054 |
| Glu | 0.038211 | 0.653019 | 0.708992 |
| Gly | 0.138395 | 0.005479 | 0.018928 |
| His | -0.05071 | 0.181659 | 0.268079 |
| Ile | -0.08496 | 0.173778 | 0.26182 |
| Lys | -0.11712 | 0.047378 | 0.100397 |
| Met | -0.0824 | 0.169483 | 0.260216 |
| Orn | 0.128469 | 0.032433 | 0.077028 |
| Phe | -0.09813 | 0.026383 | 0.065742 |
| Pro | -0.09863 | 0.101841 | 0.175908 |
| Ser | 0.156652 | 0.003582 | 0.013278 |
| Thr | 0.053131 | 0.397954 | 0.487814 |
| Trp | -0.1665 | 0.005072 | 0.018356 |
| Tyr | -0.22571 | 0.000213 | 0.001905 |
| Val | -0.09341 | 0.137866 | 0.222933 |
| ADMA | 0.075767 | 0.41675 | 0.499194 |
| alpha-AAA | -0.08452 | 0.252756 | 0.352468 |
| Creatinine | -0.03695 | 0.300202 | 0.403811 |
| DOPA | 0.022499 | 0.242466 | 0.341248 |
| Histamine | 0.011217 | 0.486119 | 0.565365 |
| Kynurenine | -0.22692 | 0.002004 | 0.009519 |
| Putrescine | -0.19783 | 0.129244 | 0.213534 |
| Sarcosine | -0.07773 | 0.269755 | 0.366096 |
| Serotonin | 0.192176 | 0.41709 | 0.499194 |
| Spermidine | -0.0233 | 0.77398 | 0.828486 |
| Spermine | -0.10946 | 0.383158 | 0.473496 |
| t4-OH-Pro | 0.069449 | 0.498767 | 0.571292 |
| Taurine | 0.076008 | 0.360454 | 0.45513 |
| SDMA | -0.0202 | 0.712498 | 0.768082 |
| lysoPC a C16:0 | -0.11745 | 0.056944 | 0.113888 |
| lysoPC a C16:1 | -0.1655 | 0.038741 | 0.086598 |
| lysoPC a C17:0 | -0.09024 | 0.20784 | 0.298035 |
| lysoPC a C18:0 | -0.16961 | 0.006948 | 0.023469 |
| lysoPC a C18:1 | -0.09007 | 0.196845 | 0.284957 |
| lysoPC a C18:2 | -0.05239 | 0.515096 | 0.584288 |
| lysoPC a C20:3 | -0.2628 | 0.00019 | 0.001855 |
| lysoPC a C20:4 | -0.21139 | 0.002801 | 0.012166 |
| lysoPC a C24:0 | -0.17805 | 0.001305 | 0.006398 |
| lysoPC a C26:0 | -0.14224 | 0.038141 | 0.086529 |
| lysoPC a C26:1 | -0.03747 | 0.5341 | 0.601357 |
| lysoPC a C28:0 | -0.02769 | 0.610444 | 0.677281 |
| lysoPC a C28:1 | -0.04982 | 0.341304 | 0.443404 |
| PC aa C24:0 | -0.04835 | 0.620996 | 0.683995 |
| PC aa C28:1 | -0.0776 | 0.078925 | 0.148105 |
| PC aa C30:0 | -0.05491 | 0.481251 | 0.565365 |
| PC aa C32:0 | -0.10827 | 0.025435 | 0.064435 |
| PC aa C32:1 | -0.22449 | 0.040699 | 0.089656 |
| PC aa C32:3 | 0.073184 | 0.257072 | 0.352544 |
| PC aa C34:1 | -0.06227 | 0.050935 | 0.104624 |
| PC aa C34:2 | -0.053 | 0.089124 | 0.158428 |
| PC aa C34:3 | 0.160263 | 0.031542 | 0.076101 |
| PC aa C34:4 | -0.09814 | 0.328242 | 0.433851 |
| PC aa C36:0 | -0.48316 | 0.000952 | 0.005361 |
| PC aa C36:1 | -0.1873 | 0.001043 | 0.00566 |
| PC aa C36:2 | -0.0519 | 0.094641 | 0.165349 |
| PC aa C36:3 | -0.06616 | 0.089637 | 0.158428 |
| PC aa C36:4 | -0.08063 | 0.02047 | 0.053646 |
| PC aa C36:5 | -0.05945 | 0.637179 | 0.696771 |
| PC aa C36:6 | -0.11475 | 0.25745 | 0.352544 |
| PC aa C38:0 | -0.21511 | 0.000061 | 0.000779 |
| PC aa C38:3 | -0.26235 | 0.00002 | 0.000475 |
| PC aa C38:4 | -0.20455 | 0.00005 | 0.000762 |
| PC aa C38:5 | -0.15959 | 0.017583 | 0.047727 |
| PC aa C38:6 | -0.17834 | 0.003083 | 0.013019 |
| PC aa C40:2 | -0.1852 | 0.000783 | 0.005162 |
| PC aa C40:3 | -0.16249 | 0.000815 | 0.005162 |
| PC aa C40:4 | -0.26274 | 0.000004 | 0.00022 |
| PC aa C40:5 | -0.26136 | 0.000036 | 0.000691 |
| PC aa C40:6 | -0.27754 | 0.000007 | 0.000229 |
| PC aa C42:0 | -0.09856 | 0.048217 | 0.100397 |
| PC aa C42:1 | -0.13434 | 0.007265 | 0.024006 |
| PC aa C42:2 | -0.16326 | 0.003481 | 0.013278 |
| PC aa C42:4 | -0.177 | 0.000457 | 0.003473 |
| PC aa C42:5 | -0.10897 | 0.056449 | 0.113888 |
| PC aa C42:6 | -0.12348 | 0.013549 | 0.039261 |
| PC ae C30:0 | -0.01079 | 0.839953 | 0.874472 |
| PC ae C30:1 | -0.00057 | 0.996121 | 0.996121 |
| PC ae C30:2 | -0.12501 | 0.007723 | 0.024457 |
| PC ae C32:1 | -0.16368 | 0.000414 | 0.003308 |
| PC ae C32:2 | -0.17206 | 0.00086 | 0.005231 |
| PC ae C34:0 | -0.21407 | 0.000617 | 0.004469 |
| PC ae C34:1 | -0.11042 | 0.033684 | 0.078768 |
| PC ae C34:2 | -0.17205 | 0.002389 | 0.011004 |
| PC ae C34:3 | -0.18637 | 0.00128 | 0.006398 |
| PC ae C36:0 | -0.21061 | 0.000058 | 0.000779 |
| PC ae C36:1 | -0.16027 | 0.003308 | 0.013233 |
| PC ae C36:2 | -0.05475 | 0.319198 | 0.425598 |
| PC ae C36:3 | -0.21645 | 0.000099 | 0.001152 |
| PC ae C36:4 | -0.33956 | 0.000000 | 0.000003 |
| PC ae C36:5 | -0.26507 | 0.000008 | 0.000229 |
| PC ae C38:0 | -0.18846 | 0.021596 | 0.055638 |
| PC ae C38:2 | -0.06111 | 0.352852 | 0.450701 |
| PC ae C38:3 | -0.17902 | 0.001165 | 0.006105 |
| PC ae C38:4 | -0.24605 | 0.000004 | 0.00022 |
| PC ae C38:5 | -0.20443 | 0.000045 | 0.000762 |
| PC ae C38:6 | -0.25449 | 0.000022 | 0.000475 |
| PC ae C40:1 | -0.24675 | 0.000267 | 0.002253 |
| PC ae C40:2 | -0.11884 | 0.016756 | 0.046306 |
| PC ae C40:3 | -0.11692 | 0.008275 | 0.025671 |
| PC ae C40:4 | -0.14078 | 0.002599 | 0.01162 |
| PC ae C40:5 | -0.16092 | 0.000925 | 0.005361 |
| PC ae C40:6 | -0.20463 | 0.000195 | 0.001855 |
| PC ae C42:1 | -0.12791 | 0.007721 | 0.024457 |
| PC ae C42:2 | -0.19274 | 0.000182 | 0.001855 |
| PC ae C42:3 | -0.18928 | 0.000692 | 0.004781 |
| PC ae C42:4 | -0.10132 | 0.037232 | 0.085747 |
| PC ae C42:5 | -0.07473 | 0.070259 | 0.135181 |
| PC ae C44:3 | -0.13144 | 0.003502 | 0.013278 |
| PC ae C44:4 | -0.10685 | 0.013778 | 0.039261 |
| PC ae C44:5 | -0.08071 | 0.111573 | 0.188434 |
| PC ae C44:6 | -0.07894 | 0.117624 | 0.19647 |
| SM (OH) C14:1 | -0.1209 | 0.0086 | 0.026145 |
| SM (OH) C16:1 | -0.12261 | 0.018333 | 0.048888 |
| SM (OH) C22:1 | -0.13697 | 0.003197 | 0.013135 |
| SM (OH) C22:2 | -0.08566 | 0.062517 | 0.123411 |
| SM (OH) C24:1 | -0.09234 | 0.047344 | 0.100397 |
| SM C16:0 | -0.08567 | 0.048143 | 0.100397 |
| SM C16:1 | -0.06946 | 0.108 | 0.18445 |
| SM C18:0 | -0.09018 | 0.085633 | 0.156017 |
| SM C18:1 | -0.07876 | 0.15517 | 0.243153 |
| SM C20:2 | 0.005589 | 0.915904 | 0.928116 |
| SM C24:0 | -0.13109 | 0.00522 | 0.018451 |
| SM C24:1 | -0.05445 | 0.223523 | 0.317527 |
| SM C26:0 | -0.01003 | 0.834018 | 0.874281 |
| SM C26:1 | -0.04203 | 0.415769 | 0.499194 |
| C0 | -0.07955 | 0.178036 | 0.265309 |
| C10 | 0.163085 | 0.07354 | 0.139726 |
| C12 | 0.161522 | 0.08622 | 0.156017 |
| C14:1 | 0.044389 | 0.49988 | 0.571292 |
| C14:1-OH | 0.109608 | 0.079961 | 0.148221 |
| C16 | -0.01415 | 0.846078 | 0.874856 |
| C16-OH | 0.015389 | 0.829761 | 0.874281 |
| C16:1 | 0.121186 | 0.144868 | 0.229375 |
| C18 | -0.03284 | 0.594363 | 0.664288 |
| C18:1 | 0.114757 | 0.166537 | 0.258302 |
| C18:2 | 0.013409 | 0.867958 | 0.885433 |
| C2 | 0.099347 | 0.34876 | 0.449251 |
| C3 | -0.0704 | 0.337465 | 0.442196 |
| C3-DC (C4-OH) | -0.02474 | 0.828245 | 0.874281 |
| C4 | -0.12488 | 0.06679 | 0.130155 |
| C5 | 0.073972 | 0.365302 | 0.45513 |
| C5-DC (C6-OH) | -0.18421 | 0.027845 | 0.068265 |
| C5-OH (C3-DC-M) | -0.18144 | 0.013948 | 0.039261 |
| C6 (C4:1-DC) | -0.00035 | 0.995711 | 0.996121 |
| C7-DC | 0.219795 | 0.010715 | 0.031935 |
| C8 | 0.07458 | 0.364043 | 0.45513 |
| C9 | 0.01704 | 0.85546 | 0.87858 |

S2 Legend. Delta was calculated by log2 transformation of metabolite expression data (μM), computing the difference between post-treatment and baseline for each patient, and computing the average of that difference separately for PH and control groups.

**S3. Treatment effect sizes before and after controlling for baseline metabolite levels**


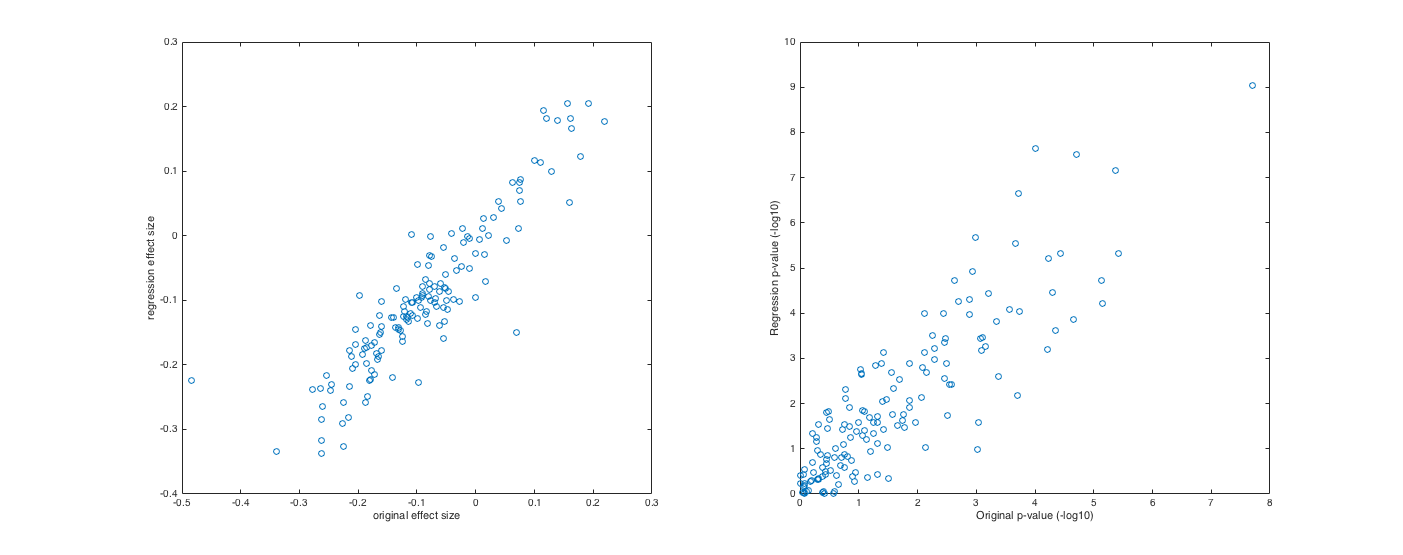


S3 Legend. As stated in the manuscript, three of the 12 original metabolites dropped out after controlling for baseline metabolite levels and 9 additional metabolites were identified (Bonferroni correction, adjusted *p*<0.01). However, this overstates the significance of the changes. The graphs below show that both statistical significance and the direction of the identified effects were very similar with both analyses. The x-axis of Panel 1 (left) shows the effect size before controlling for baseline metabolite levels and the y-axis shows the effect size after controlling for baseline metabolite levels. The x-axis of Panel 2 (right) shows the *p*-value before controlling for baseline levels and y-axis shows the *p*-value after controlling for baseline levels.

**S4. Box plots showing key metabolite changes after controlling for baseline metabolite levels in multiple regression (adjusted *p*<0.01, Bonferroni corrected)**


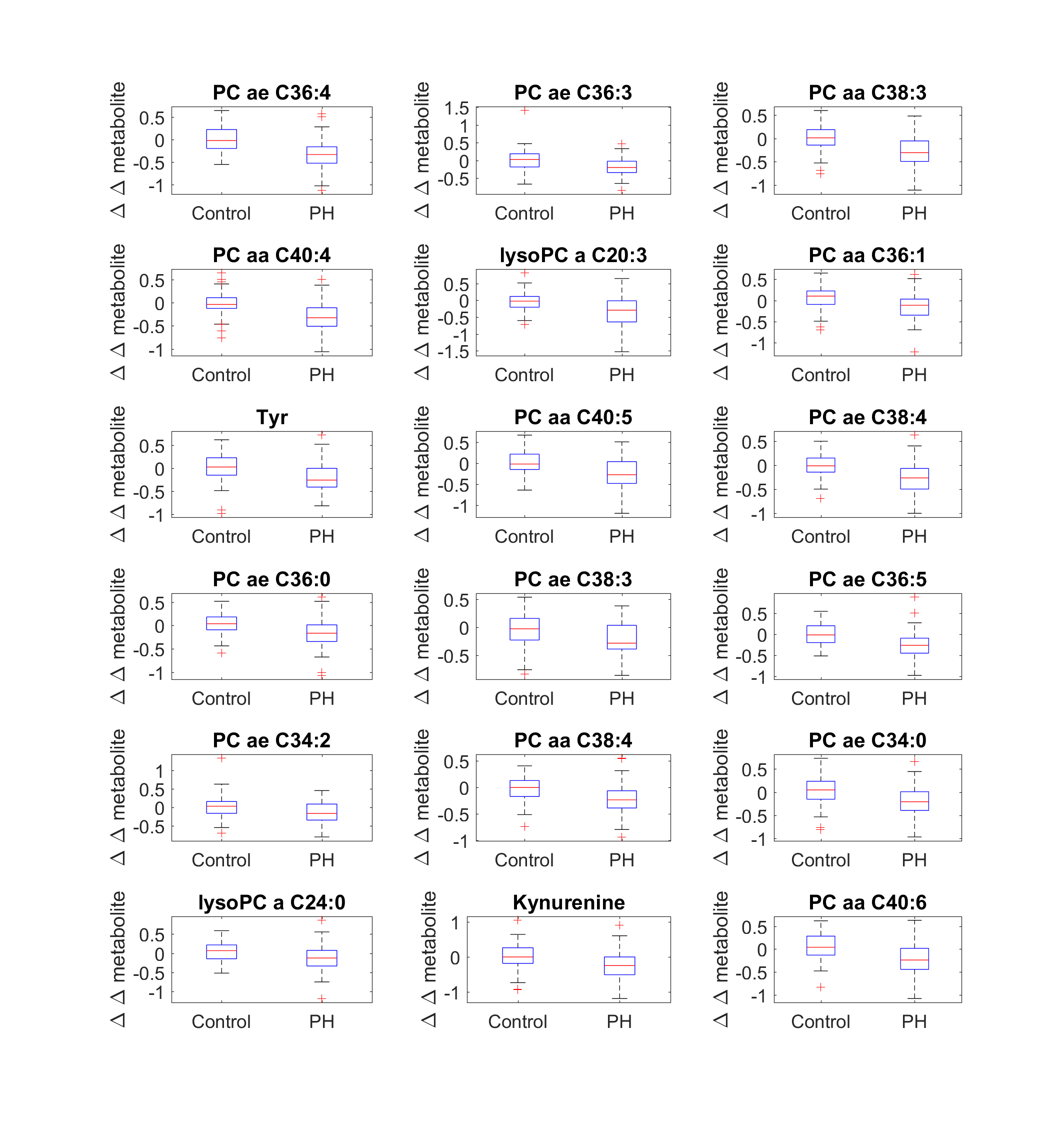


S4 Legend. Boxplots display change in log2 expression from baseline to post-intervention for the Control and Perfect Health (PH) groups. Delta was calculated by log2 transformation of metabolite expression data (μM), computing the difference between post-treatment and baseline for each patient, and computing the average of that difference separately for PH and control groups. Bars display standard deviation.

**S5.** **Quality control data.**


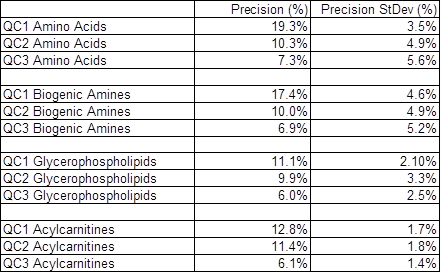


**S6**. **Principal components analysis**.


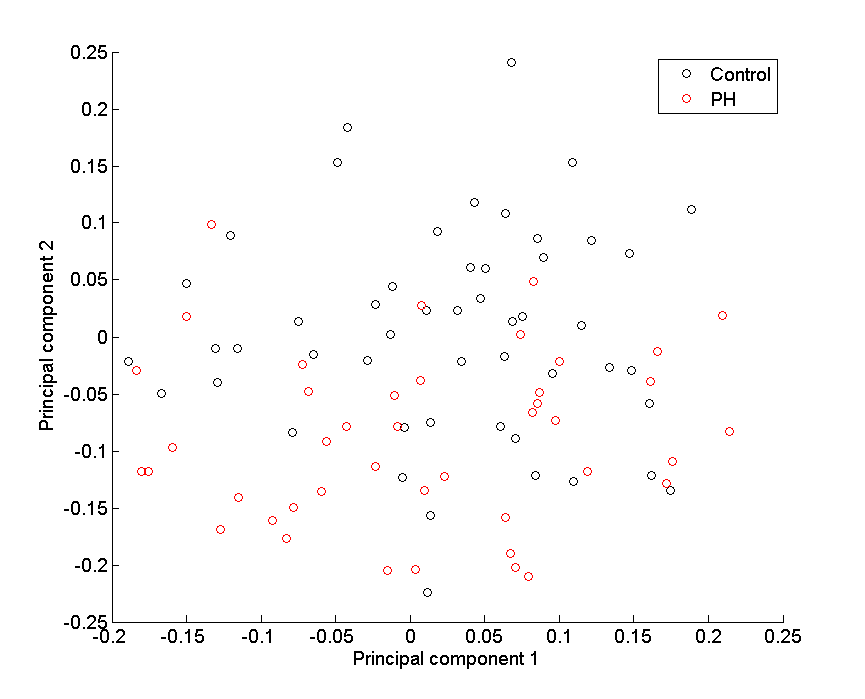


S6 Legend. Principal components 1 and 2 are plotted against each other. There is a statistically significant difference in principal component 2.
